# Atmospheric pressure plasma jet for respiratory face masks decontamination and re-use: Considerations on microbiological efficacy, material impact and product lifecycle

**DOI:** 10.1371/journal.pone.0313041

**Published:** 2025-01-23

**Authors:** Diletta Scaccabarozzi, Jessica Ponti, Sabrina Gioria, Dora Mehn, Taija Sinkko, Fulvio Ardente, Francesco Fumagalli

**Affiliations:** European Commission, Joint Research Centre (JRC), Ispra, Italy; Kwangwoon University, REPUBLIC OF KOREA

## Abstract

Disposable filtering face piece respirators (FFRs) are not approved for reuse as standard of care. However, lessons learnt from the SARS-CoV-2 pandemic, FFRs decontamination and reuse may be needed as crisis capacity strategy to ensure availability in medical facilities. We studied a decontamination methodology based on atmospheric pressure plasma technology, which allows for rapid, contact-free decontamination without utilisation of harmful chemicals, and suitable to access small pores and microscopic filters openings. Promising performances in terms of bioburden reduction (Log6) were achieved while imparting mainly transient chemical surface modifications to the masks filtering layers. The plasma decontamination process proposed was also considered in terms of the environmental impact of re-use technology for FFR medical devices in order to understand its sustainability. This study assessed the feasibility of an atmospheric pressure plasma approach for the decontamination of disposable filtering face piece respirators (FFR) or respiratory masks commonly used in hospital settings.

## Introduction

The pandemic of coronavirus disease 2019 (COVID-19) caused by severe acute respiratory syndrome coronavirus 2 (SARS-CoV-2) infection has resulted in an unprecedented setback for global economy and health [1]. On January 2024, according to the World Health Organization’s (WHO), the COVID-19 pandemic has reached 773 million reported infection cases and over 7 millions confirmed fatalities [2]. The scale of the disease and its quick spread, especially in the first phases with no access to vaccines, proved to be an extremely serious strain to public health organisations raising concerns about their resilience and preparedness.

Person-to-person viral transmission occurs mainly via respiratory droplets containing viral particles generated by sneezing or coughing. Such aerosol can transmit the virus either by indirect routes (larger droplets depositing on surfaces) or airborne routes (smaller particles travelling in the air) [3]. In the early stages of the pandemic, medical authorities have identified the use of properly fitted medical face masks (FPP2 or FPP3 European standards or equivalent like N95 –U.S.-, KN95, -China-, P2 –Australia-, DS–Japan-) as one of the best practices to prevent and slow down transmission. Their use has become so ubiquitous and central in our society that masks are now perceived in popular culture as one of the objects symbolising the pandemic.

Face masks are normally made from synthetic thermopolymers (mainly polypropylene, although other polymers like polycarbonate and polyesters can be used) and generally rely on a three layers structure to collect and block aerosol particles with different sizes [4]. Standard arrangement comprises an inner part based on soft fibres, an intermediate layer acting as main filter and an outer layer, the latter based on non-woven fabric with hydrophobic properties. Polypropylene is an industry standard as masks filtration layer material due to its low adhesion characteristics, humidity repellence, good mechanical properties and cost effectiveness.

Masks surfaces may become contaminated during exposure to pathogen-containing aerosols. In the case of SARS-CoV-2, the stability of the virus on plastic and metal surfaces has been reported lasting up to 72 hours [5], thus enforcing a strict single-use practice of disposable FFR.

As early as January 2020, just at the beginning of the COVID-19 crisis, face masks were the only known way to reduce infection risk and demand rose abruptly. To avoid supply bottlenecks, crisis mitigation practices attempted to rationalise the use of masks in both private life and healthcare environments [6]. However, the quick spread of the pandemic and the just-in-time supply chain structure of hospital commodities (that included face masks) determined a personal protective equipment supply crisis, acute market competition and spiralling prices. More importantly, shortages in masks supply capacity increased contagion risk among healthcare personnel and, in general, generated a less effective response to the healthcare crisis [7]. Medical face masks are currently not approved for routine decontamination either in EU or in US. However, in the emergency situation created in many countries, lead to consider mask decontamination and reuse as a short-term response to ensure continued masks availability. Moreover, long-term environmental sustainability considerations about single-use, plastic-made medical face masks usage during the pandemic also highlight the need for a re-use approach. WHO estimated that, during the peak 2 years of the pandemic event, about 89 million FFR units worldwide would have been needed each month just for protecting health workers, therefore it was calculated that mask production should have been increased by 40% of the total annual production [7]. These actions would have resulted in a steep increase in volume of biomedical hazardous waste to be treated, which often means incinerated, and a corresponding steep increase in CO_2_ emissions from this process. Thinking even further forward, usage of disposable face masks as a public mass protection strategy may easily increase societal monthly consumption in the billion-units range, in this case a substantial fraction of these items are likely to enter the ecosystem as litter and contribute to the raising threat of environmental micro-nano plastic pollution.

One approach to mitigate both short and long-term issues related to medical masks could have been the implementation of the decontamination and reuse protocols although a reference procedure, approved by medical authorities, containing standard rules for re-use is still missing. Ideally such a strategy would show (i) decontamination effectiveness, (ii) preservation of mask materials properties (filtration efficiency) and (iii) absence of residual health hazards after processing. A common approach during the supply crisis in 2020 was to retrofit and deploy existing decontamination methods and apply them to masks. None of these works tested disinfection directly against SARS-CoV-2 but used, as proxy, less dangerous influenza viruses of lipid bilayer enveloped virus), bacteriophages (e.g. MS2) or bacterial spores (e.g. B. Subtilis, Geobacillus Stearothermophilus) to validate decontamination data for COVID-relevant scenarios. Masks material physical chemical properties degradation as function of decontamination cycles are in comparison less reported. Several works [8–10] compared results of the most investigated approaches. In the majorities of the cases, proposed protocols, whilst efficient in eliminating the contamination [11–13], as standard autoclaving [10], dry heat [14], moist heat treatment [15–17], UV-C irradiation [18], ethylene oxide (EtO) [14, 15, 19, 20], or hydrogen peroxide (HPG) [3, 14, 15, 19] gassing, they all resulted in affecting masks functionalities at a certain degree. The rationale of the existing body of literature suggests that moist heat and HPG approaches show a preponderance of benefits over harms for mask decontamination cycling and these ideas were de-facto adopted and incorporated in several recommendations (CDC, FDA) about emergency re-use of protective face masks in hospitals.

Several attempts were done to assess plasma discharges as tools for mask decontamination [21–24]. Atmospheric pressure plasma sources have been vastly investigated for medical applications, notably in the sub-field of disinfection and sterilisation for heat-sensitive, non-flat geometry substrates [25, 26]. Interest in plasma technology for masks decontamination stems from its toxic-chemicals free approach, from the possibility of producing highly reactive chemical species keeping low temperature at the application point and from safe and simple handling, thus making it an interesting alternative for processing heat-sensitive medical devices and it is suitable to rapidly access even small pores and microscopic openings typical of FFR filter materials. APPJ effectiveness for decontamination was already demonstrated against different bacterial strains over complex surfaces like agar medium [27], catheters [28] and food surfaces [29, 30]. Specific studies employing atmospheric plasmas for human viruses decontamination over different surfaces employed enteric viruses such as norovirus, adenovirus and hepatitis A, treated in water or liquid media [31–33], organic substrates such as food [34, 35] and inorganic such as steel [36] or glass [37]. Atmospheric plasma decontamination over hard surfaces approach was also tested against respiratory viruses such as flu [38] or SARS coronaviruses [39].

In this work, we investigate an atmospheric-pressure plasma technology approach for the decontamination of contaminated face mask surfaces. We report the successful inactivation of different biological models (from bacteria to viruses and bacterial spores) used as proxy for SARS-CoV-2, directly on contaminated face masks. In addition, the material chemical-physical properties of masks filtering layers were analysed before and after repeated treatments and we observe that the sterilisation process does not substantially modify material physical chemical characteristics. Finally, we include considerations about the environmental impact of single use face masks against plasma decontamination and re-use, identifying key-technological aspects that should be modified in order to make the decontamination process less environmentally impactful. The environmental analysis and life cycle assessment of plasma decontaminated face mask are thoroughly investigated in a dedicated, parallel publication [40].

## Materials and methods

### Filtering face piece respirators models

Four commercially mask models were used in the study. All biological tests were executed on FFP2 “ARIA + Class 2001 Covid-19” masks models, in compliance with EU regulation n. 2016/425 and PPE-R/02.075 Ver. 2; all masks used are CE certified by MTIC INTERCERT S.r.l–Notified body n. 0068 (Milan, Italy). This model of face masks consists of 5 polypropylene based non-woven layers. Masks material degradation tests were also executed on two other FPP3, namely FFP3 AS330310 (CE 0426) and FFP3 839V (CE 012), and one standard surgical mask type. The masks were cut in pieces measuring 1.5 X 1.5 cm and sterilised by autoclaving at 121°C and 1.1 bar for 20 minutes before the use.

*Plasma source*: for the experiments a COST- Plasma jet was employed. It is an atmospheric pressure plasma jet (APPJ) developed as reference source for the medical and biological applications of non-thermal plasmas [41, 42]. COST—APPJ is non-thermal, capacitively-coupled plasmas operated with RF power (P_abs_ ≈ at 13.56 MHz), with two parallel stainless steel electrodes separated by 1 mm discharge gap and operating in remote, cross-flow configuration. Typical gas mixture used is He (1 slm) with 0.5% O_2_. Typical electrode–substrate distance was 2 mm. Current–Voltage waveforms were monitored via a Tektronix MD034 mixed domain oscilloscope and TPP0250 10x passive probes.

### Biological models

*Escherichia coli K12*: *Escherichia coli* (Migula) Castellani and Chalmers (ATCC 29425) were grown in Luria Bertani (LB) Broth, Miller (BD Difco Laboratories, Detroit Mi) to saturation overnight at 37°C with agitation (300 rpm). Cells were harvested and resuspended at 8,7x10^8^ CFU/mL in LB broth (the concentration of the bacteria were determined by OD600 measurement). *Bacteriopahges MS2* (ATCC 15597-B1) were used to evaluate the antiviral activities of the plasma as a potential surrogate of SARS-CoV-2. The propagation protocol is described in the next paragraph. *Pseudovirus*: Spike Pseudotyped Lentivirus (78206, BPS Biosciences) was used according to BPS Bioscience protocol and green fluorescent protein (GFP) was measurement after 48 hours of incubation at 37°C. *Geobacillus stearothermophilus*: *Geobacillus stearothermophilus* (ATCC 7953) spores inoculated on stainless-steel coupons in predefined concentrations (1–5 x 10^6^ CFU/coupon, from Liofilchem Diagnostic) were treated or not with plasma for 15 minutes and observed by Scanning Electron Microscopy (SEM). *A549 alveolar epithelial cells* (ATCC CRM-CCL-185): the cells were handled and subculture following the manufacturer’s instructions.

### Bacterial contamination protocol

A volume of 5 μL of *E*.*coli* microbial suspension (8,7x10^8^ CFU/mL) were spotted on masks. In order to increase permeability/hydrophilicity of the fibre, the masks were pre-treated with glow discharge (10 mA, 150 sec).

Contaminated FFRs were exposed to plasma source and treated for increasing times (from 5 to 15 minutes). After plasma treatment, the masks were incubated in 5 mL of LB Broth and vortexed for 1 minute. A volume of 100 μL from each sample was then plated in agar dishes in a 10-folders serial dilution. The agar dishes were incubated for 24 hours at 37°C. At the end of the incubation time, colonies formed were counted and the number of CFUs recovered from the mask were calculated (mean ± SD).

To determinate the antibacterial effect of plasma, the inhibition zone of the growth of bacteria was calculated on agar plates after plasma treatment. In detail, on LB Agar plates, 100 μL of *E*.*coli* were plated at 8,7x10^8^ CFU/mL and left to rest for 10 minutes. Then the plates were treated or not with plasma for 300 sec/600 sec and 900 sec. Plates were incubated overnight at 37°C and then imaged. Zones of inhibition were quantified using the GelCount analyser. The results are expressed as mean ± SD of 10 experiments run in quadruplicate.

### Bacteriophages propagation

The host strain *Escherichia coli* (ATCC 53323) was grown statically at 37°C for 6 hours. The growth media (ATCC, M271 medium) contained 10 g tryptone, 1 g yeast extract, 8 g NaCl/L was autoclaved, then supplemented with glucose (0.1%), CaCl_2_ (2 mmol/mL) and thiamine (10 g/mL). Bacteriophage MS2 was propagated in the exponentially growing *E*. *coli* culture overnight (16 h) at 37°C. After the incubation, the bacteriophages were purified by a centrifugation step at 3000 *g* for 10 min and by filtration using a 0.22 μm syringe filters (Millipore) to remove the bacterial cells and debris.

Phage suspension stocks were prepared with a titer of 10^11^ to 10^12^ PFUs/ml and stored at 4°C.

### Mask contamination with MS2 bacteriophages and double layer plaque assay

A volume of 5 μL of bacteriophages suspension at 1x10^7^ PFUs/mL were spotted on pieces of mask measuring 1.5 X 1.5 cm. The concentration used was defined to mimic COVID-19 droplet transmission after coughing or sneezing [43].

Otherwise differently specified in the text, plasma discharge parameters used in for the decontamination experiments were: He/O_2_ gas mix (O_2_ 0.5%, total flow 1 slm), total RF power of 1.4W, for 15 minutes, at a distance of 2 mm. After plasma treatment, the pieces of masks were vortexed for 3 minutes in 5 mL of M271 medium and 10-folder diluted in PBS. A volume of 10 μL of each sample were placed into two-layer agar plates and incubated for 24 h at 37°C following the protocol described by Sambrook [44]. Grown plaques were counted and converted into log reduction. Data are expressed as mean ± S.D. of 5 experiments run in quadruplicate.

### MTT test

MTT assay was performed on A549 alveolar epithelial cells (A549 cells; ATCC CRM-CCL-185) to evaluate the cell viability after plasma treatment. A549 cells were plated in 96-well cell culture plates (Corning Inc., Corning, NY, USA) at a density of 10 × 10^4^ cells/well and allowed to adhere for 24 h, then exposed to plasma treatment for 15 minutes. Medium control and a positive control (Triton 0.1%) were included in each assay. At the end of the exposure time, the supernatant was removed. Cell viability was evaluated using MTT [3-(4,5-dimethylthiazol-2-yl)-2,5-diphenyl-2H tetrazolium bromide] (Sigma-Aldrich, Inc.) added to the cells in fresh complete culture medium at a final concentration of 250 μg/mL. After 4 h of incubation at 37°C the supernatant was removed and the precipitated formazan crystals were dissolved in 200 μL DMSO (Sigma-Aldrich, Inc.) followed by 25 μL of glycine buffer (0.1 M glycine with 0.1 M NaCl). The absorbance was quantitated at 570 nm by the EnSpire® Multimode plate reader (Perkin Elmer, Milano-Italy-) using a reference wavelength of 680 nm. Data are expressed as percent of mitochondrial activity, and reported as mean ± SD. Three independent experiments were performed in triplicates.

### Electron microscopy analysis

To investigate the MS2 size and shape, the bacteriophages suspension was analysed by transmission electron microscopy (JEOL JEM 2100, Jeol, Italy). A volume of 3 μL of each suspension was manually deposited on 200 mesh Cu-Fomvar-Carbon coated grid (TEDPELLA, Inc, USA), left to dry overnight in a desiccator. Samples were then positively stained with Uranyl acetate (Sigma Aldrich, Italy) 5% (w/v) for 3 minutes, dried and analysed at 120 KV. The bacteriophages’ min Feret was manually measured on 300 single bacteriophages by Image-J, and primary mean size calculated by Gaussian fitting. Mask fibres (i) untreated as control, (ii) infected with MS2 and (iii) infected with MS2 followed by 15 minutes plasma treatment were gently detached with tweezers and deposited on 200 mesh Cu-Fomvar-Carbon coated grid (TEDPELLA, Inc, USA) where 5 μL drop of MilliQ water was previously deposited. Grids were left to dry and analysed as described above.

### Scanning electron microscopy analysis

*E*.*coli* K12 bacteria were deposited to a cleaned silicon surface (20, 40, or 60 μL), dried at 37°C and treated or not for 15 minutes with plasma. For SEM studies JEOL-JEM 2100 microscope, made in Japan, was used.

### Fourier transformer IR spectroscopy

FTIR-attenuated total reflectance (ATR) was exploited to characterise the chemistry of mask non-woven layers before and after plasma treatment. The measurements were performed in ATR mode, the measured area was reduced via masking slits to 25 μm x 25 μm, acquired spectral range was 600–4000 cm−1; spectral resolution used was 2.0 cm−1; every spectrum was accumulated 258 times. The analyses were carried out using Bruker Hyperion μ-FTIR spectrometer with integrated microscope equipped with a combined 20x Cassegrain/ATR (Germanium crystal, diameter 100 μm, refractive index n = 4.0) objective. OPUS software (native Bruker data analysis environment) and OriginPro9.0 were used for data processing.

### Optical emission spectroscopy

OES allows to monitor the stability and reproducibility of APPJ operation and the influence of composition and impurities within the discharge gas mix. To characterise the delivered plasma chemistry for a given process parameters setup, we record plasma emission with a broad band spectrometer (Avantes) coupled with fibre optics.

### Statistical analysis

Data are expressed as mean ±  standard deviation (mean +/-SD) of at least four experiments performed at least in triplicate. Data were analysed by unpaired one-way analysis of variance (ANOVA) followed by Bonferroni post hoc test. Statistical analysis was performed using GraphPad Prism 5.0 software (GraphPad Software Inc., San Diego, CA, USA). p < 0.05 was considered statistically significant.

## Results and discussion

In the following we present and discuss our investigations on an atmospheric-pressure plasma technology approach for the decontamination of contaminated respiratory face mask surfaces. First we present a brief characterization of the plasma sources utilised for the process. In a second paragraph we present results about the decontamination efficacy of our optimised process and then we discuss the physical-chemical properties modification observed on the masks fibres comparing them before and after plasma treatment. Finally, we present a summary of our considerations about environmental impact and life cycle assessment of our proposed re-use technology for face masks medical devices as compared to emerging alternatives present in literature.

### APPJ characterization

**Fig 1:**
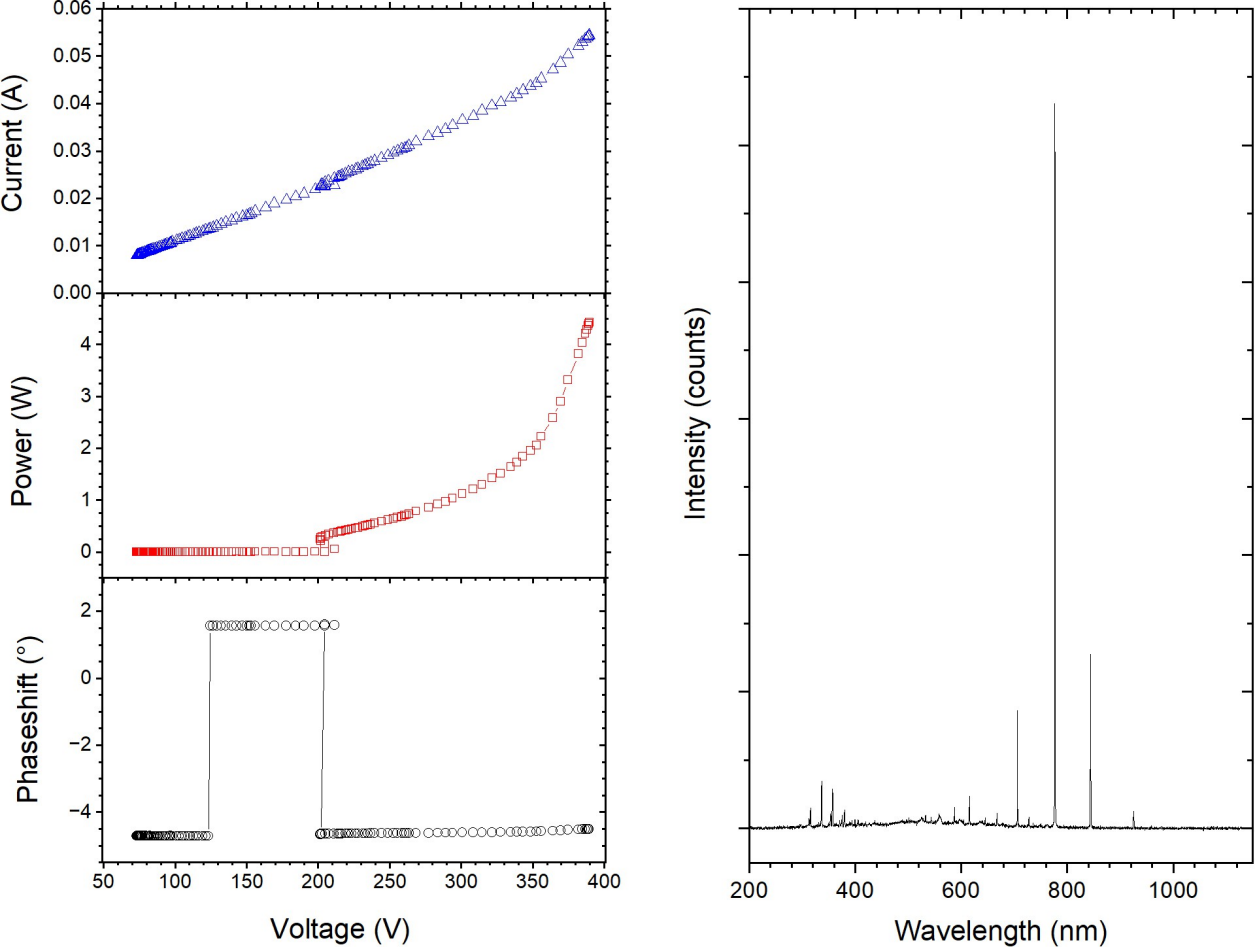
Electrical characterization (A) current (up), power (middle) and phase (low) as function of applied voltage) and optical emission spectrum (B) of the atmospheric pressure plasma jet operated in He/O_2_ (1slm).

The total power dissipated by the CoST-APPJ was measured directly using the oscilloscope via voltage and current probes (P = V·I·cos[φ]) installed within the source coupling circuit [41]. For a pure He gas feed the system ignites the discharge at around 160 V, or ≈ 200 mW and delivers a stable “glow-mode” plasma discharge up to 330 V or ca. ≈ 1.7 W, above this voltage a transition to constricted-mode at the electrode tip is observed (Fig 1A) During the processes the system was normally operated at 310 ± 10 V delivering 1.4 ± 0.1 W. Due to the combination of relatively high gas flow, remote operation of the discharge and small power output the decontamination process thermal load is quite limited. The FPP2 mask non-woven layers temperature, measured directly under the effluent of the plasma source at a vertical distance of 2 mm form the nozzle, raised only by 3°C during 15 min of processing.

OES (Fig 1B) diagnostics allows to monitor the production of sterilising chemistry in different regions of the plasma effluent, as well as to evaluate the stability of the plasma discharge and the occurrence of impurities. In our processing conditions, He/O_2_ plasma most prominent features in the emission spectrum are atomic oxygen lines (777 nm, 844 nm and 926 nm) and He atomic lines (706 nm, 587 nm and 668 nm). Due to effluent mixing with atmospheric air at the nozzle exit also OH (308 nm) and N_2_ 2^nd^ positive system (band head at 336 nm) and UV lines from NO molecules emission (248 nm) are also detectable (see Fig 1). Although not detected directly via OES, O_3_ production was also reported to be a possible sterilising by-product generated in our discharge conditions [45]. Electron paramagnetic resonance spectroscopy and nuclear magnetic resonance experiments on similar systems indicate that also more reactive short-lived superoxides (such as H_2_O_2_) form in the plasma effluent stream between the nozzle and the substrate. UV photons, heat and reactive oxygen (ROS), nitrogen (RNS) and nitro-oxide (RNOS) species were reported to have bactericidal effects and to contribute to the inactivation of harmful biomolecules such as mammalian viruses and bacteriophages on flat surfaces [23, 46, 47]. It is speculated that plasma produced sterilising agents can inactivate viruses by chemically modifying the capsid, the DNA (or RNA) strands or by direct structural damage [48]. However, the interaction of these plasma-produced sterilising agents and their ability to diffuse into a micro-porous medium such as the masks non-woven layers has not been investigated in detail. On the other hand, these very same physical agents can also potentially interact in a destructive way with the polymeric materials comprising the face mask and hinder their functionality.

### Process impact on mask materials

Masks items were stripped of their metal components (when present) and items sections of radius ca. 40 mm were positioned onto the grounded treatment plate. All the processing conditions tested resulted in no significant change on visual and tactile inspection. After each processing cycle, water contact angle measurements of the mask layers were performed in order to verify plasma induced modification of the outer mask layer. In both cases, before and after the treatment, the outer non-woven layer showed a highly hydrophobic character, where the water droplet failed to adhere to the surface. This behaviour showed a time transient, just after exposure to the plasma discharge the surface became wettable and the water droplets were readily absorbed from the underlying layers but it was also observed that after ca. 2 hours after the plasma treatment the surface exhibits again an hydrophobic character where water droplets have difficulties to adhere. From these tests, it was then concluded that the contact angle was > 140° and the surface hydrophobic character of the surface was substantially unchanged.

**Fig 2:**
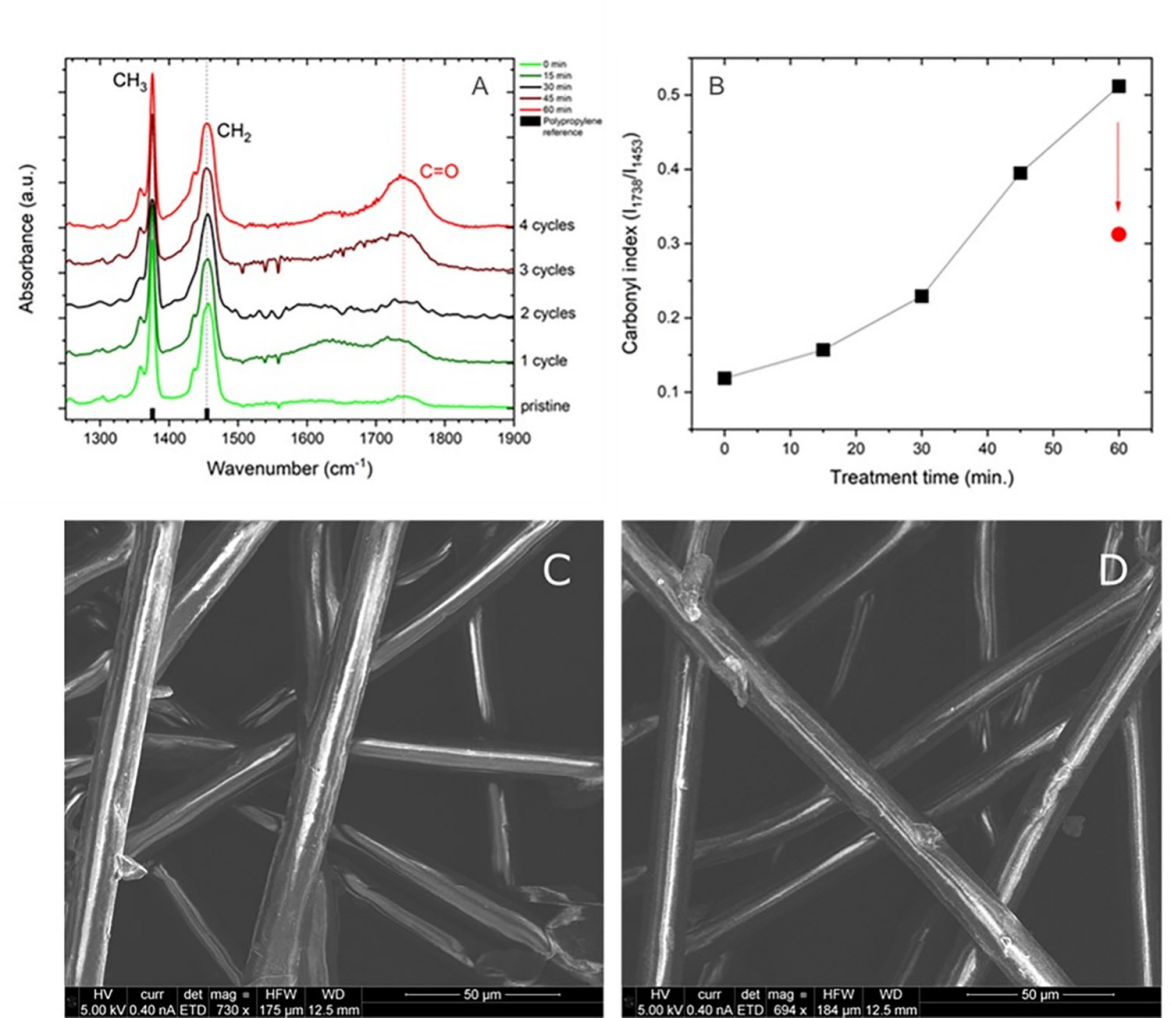
ATR-FTIR spectra of the respiratory masks filter materials before and after several cycles of plasma treatment (15 min). First consecutive treatments were executed with a 15 minutes time delay between each other, after four cycles the mask materials were stored overnight and the next treatment was executed after 24h. Panel a) IR spectra fingerprint region between 1250 and 1900 cm-1, panel b) carbonyl index, calculated as the intensity ratio of C = O stretching at 1738 cm^-1^ and CH2 bending at 1453 cm^-1^. c-d) SEM images of outer mask non-woven layer before (c) and after (d) plasma exposure.

FTIR spectroscopy in contact mode (ATR) was used to characterise surface chemistry modifications induced by plasma decontamination process. Prior to exposure to plasma, the different mask layers were analysed and the spectral fingerprints of polypropylene resulted in the most likely assignments for the filtering non-woven layers (see S1 Fig in S1 File). Plasma treatment did not induce large modifications in the spectral fingerprint but after exposure the growth of a broad shoulder at 1720–1760 cm^-1^ can be seen (see Fig 2A). Vibrations in this range can be attribute to a carbonyl (C = O) stretching and it is compatible with the grafting onto the polymeric fibres of O_2_* or super-oxides species generated by the plasma source. The ratio between the carbonyl peak and the methylene (CH_2_) bending vibration peak at 1453 cm^-1^ can be used to qualitatively characterisethe O species grafting on the mask surface. (I _1738_ / I _1453_) peak ratio as a function of plasma treatment time is shown in Fig 2B. The amount of grafted oxygen steadily increases after consecutive treatment cycles with the ratio increasing roughly 4-fold after four cycles. In this case, the delay between consecutive treatments was 10 minutes. After additional 6 hours though, the peak ratio was measured again and resulted in an average value of 0.3 (decreased to ca. 60% as compared with the value measured after the last plasma cycle) suggesting the transient nature of the grafted oxygen functionalities. This effect, often termed “hydrophobic recovery”, is known to be time-dependent and can lead to a partial restoration of the pristine surface chemistry. It is driven by recombination with air molecules of the surface-grafted peroxides or, specifically for polymers, reorientation of the polymeric chains causing migration of the oxidised groups from the surface to the bulk.

### Biodecontamination effectiveness

Before starting optimising plasma process parameters for treatment of mask materials substrates, the plasma treatment efficacy was verified on several biological models, covering the range of realistic contamination occurring during real life use, where a technique capable of eliminating a broad class of pathogens during the same process is desirable. In this work, the capability of plasma to eliminate bioburden onto face masks layers was investigated using bacteriophages MS2 and spike pseudotyped lentivirus Sars-CoV2 as different viral models, *E*.*Coli* as a bacterial model and *Geobacillus Stearothemophilus* as a bacterial spore model. The viral models were chosen to verify the specificity of the plasma decontamination process against viral infections. Bacteriophages are a popular used model due to the following advantages: inexpensive procurement, simpler handling protocols, safety and established characterization methodologies. The pseudovirus SARS-CoV2 is a more specific proxy for SARS-CoV-2 and comprise the virion capsid and proteins without its inner mRNA. *E*. *Coli* was chosen since it represents a very known standard in the field and allows for comparisons with existing literature while the bacterial spores were tested as they represent one of the worst case scenario in terms of sterilisation resistance (much harder to eliminate with respect to viruses).

**Fig 3:**
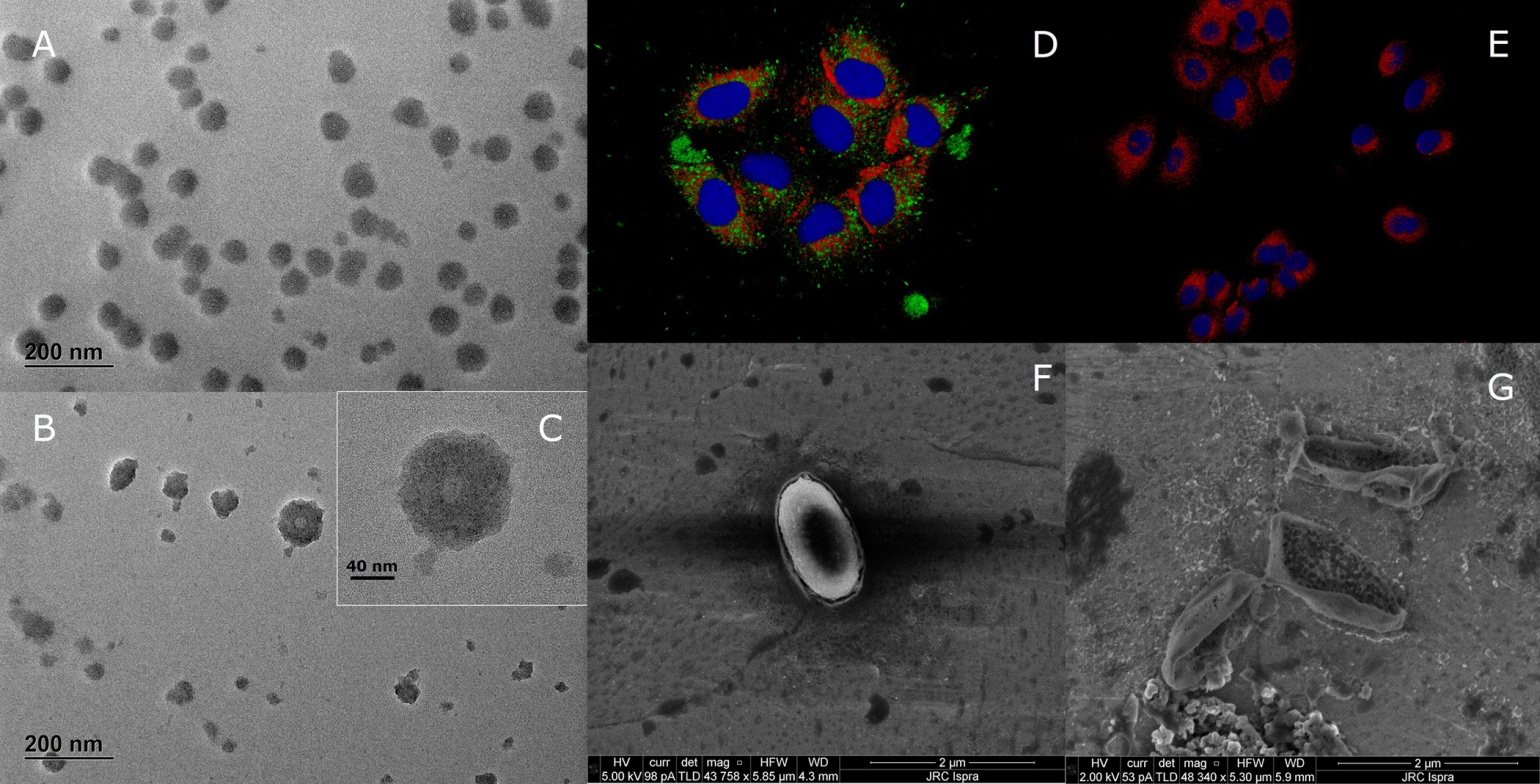
TEM images of untreated (A) and 15 minutes plasma treated MS2 bacteriophages previously deposited on Mask (B,C). Confocal fluorescence microscope images of A549 alveolar epithelial cells infected with Spike pseudotyped lentivirus Sars-CoV2 (D), not plasma treated, used as control and treated with plasma for 15 minutes (E). Colour code is red for hACE2 receptor, blue for the nuclei and green for the pseudovirus. *Geobacillus Stearothemophilus* bacterial spores prior (F) and after 15 minutes of plasma treatment (G).

TEM images (Fig 3A) show the morphology of MS2 bacteriophages (mean size of around 30 nm, estimated using Feret minimum, see S2 Fig in S1 File) after incubation on mask. Masks incubated MS2 appear more electron dense as compared to bacteriophages directly generated by bacterial incubation (S2 Fig in S1 File) and once purified and maintained in culture medium, the MS2 tend to form agglomerate and aggregates, apparently increasing their primary size. After plasma exposure, the analysis on mask fibres directly deposited on Cu-grid, that clearly show the presence of a layer surrounding the bacteriophages which is absent in the control (S3 Fig in S1 File), TEM imaging after plasma treatment reveals morphological changes in both single MS2 (Fig 3B) and agglomerates (Fig 3C) showing the inner voids of capsid structures and an increased roughness.

These morphological observations suggest the occurrence of both physical and chemical interaction with plasma reactive species. Bombardment with ROS could lead to chemical bonds breaking and erosion of various virus structures due to surface reactions with different radical species (O_2_, O, OH, etc.). Lesions and perforations observed in the lipid membrane (Fig 3C) are likely to occur due to this type of plasma biomaterials interactions. Oxidative stress could also cause protein denaturation, amino-acids oxidation and damage to RNA, all of these pathways possibly leading to a reduction in viral activity and replication efficacy. A more quantitative evaluation of the plasma treatment efficacy in inhibiting viral activity was performed by infecting A549 alveolar epithelial cells with plasma treated MS2 bacteriophages recovered from mask materials. After 24 h of incubation a cell viability test (MTT) was performed and the results compared with blank and control (i.e. no exposure to plasma treatment) samples. In our experimental conditions, results obtained show that the viability of cells incubated with bacteriophages and not plasma treated was significantly decreased (down 40% of the control value) while the viability of cells incubated with bacteriophages and treated with plasma did not statistically differ from the control value (see S4 Fig in S1 File). This supports the conclusion that after 15 minutes of plasma exposure the viruses are inactivated and not able to infect the cells thus expressing their toxicity. To further investigate plasma-induced chemical damage effects that can impair viral activity, a batch of A549 alveolar epithelial cells were infected with spike pseudotyped lentivirus Sars-CoV2 previously exposed to plasma treatment for 15 minutes and compared with a cell culture exposed to untreated control. The ability of the pseudovirus to infect the cells, was analysed with fluorescent confocal microscope (Fig 3D and 3E) to investigate the localization of the pseudo-virus (stained with GFP) within the cells. The fluorescence images show how in the case of plasma exposure the pseudoviruses (green) were not able to bind on the cell structures (red and blue in Fig 3D and 3E). The opposite behaviour is apparent in the control sample image where a substantial signal from Sars-CoV2 pseudovirus is detected in the proximity of the cells. This result supports the conclusion that even in cases where the rate of surface reactions between viruses structures and plasma-generated ROS is not high enough to lead to capside erosion and physical destruction of the virus structure, the accumulated oxidative damage to capsid proteins and membrane lipids is substantial enough to inhibit viral functions crucial for virus proliferation. Literature data on different surfaces indicate that direct plasma treatment is more effective in inactivating viruses rather than on bacteria or spores [5]. A realistic decontamination scenario would imply to remove from the masks layers all types of harmful biomolecules and microorganisms, therefore the decontamination efficacy of the plasma treatment on mask materials was verified also using bacteria and bacterial spores biological models. In Fig 3F and 3G, SEM images of spores of *Geobacillus Stareothermophilus* recovered from face masks exposed to plasma action are shown. The control sample shows an intact spore prior treatment, with an oval shape and major axis of ca. 1.8 μm length [49]. After exposure to the plasma discharge (Fig 3G), the spores suffered clear physical damage with disruption of the bacterial membrane and espouse of the inner structures.

An important parameter for the feasibility of masks (and also other objects) decontamination process is the achievable bioburden reduction. A standard Log6 reduction (6 orders of magnitude concentration reduction for a given contaminant) is normally required by sanitary SOPs in real applications independent from the type of substrate or the type of harmful biomolecule/microorganisms to be decontaminated. In parallel, it is required that such a decontamination level is reached without hampering the functionalities of the treated substrate. Considering the mask materials compatibility limitations illustrated in Fig 2 and subsequent discussion the maximal achievable bioburden reduction was evaluated exposing masks to a single decontamination run. The masks, infected with bacteria *E*.*Coli* or bacteriophages MS2, were exposed (against control) to the plasma discharge operated in He/O_2_ gas mix (O_2_ 0.5%, total flow 1slm), total RF power of 1.4W, for 15 minutes, at a distance of 2 mm. The bacteria or viruses extracted from the mask layers -using the protocol described in the methods section- were incubated for 24 hours at 37°C and the decontamination efficacy assessed with a colony forming/ plaques units measurements.

**Fig 4:**
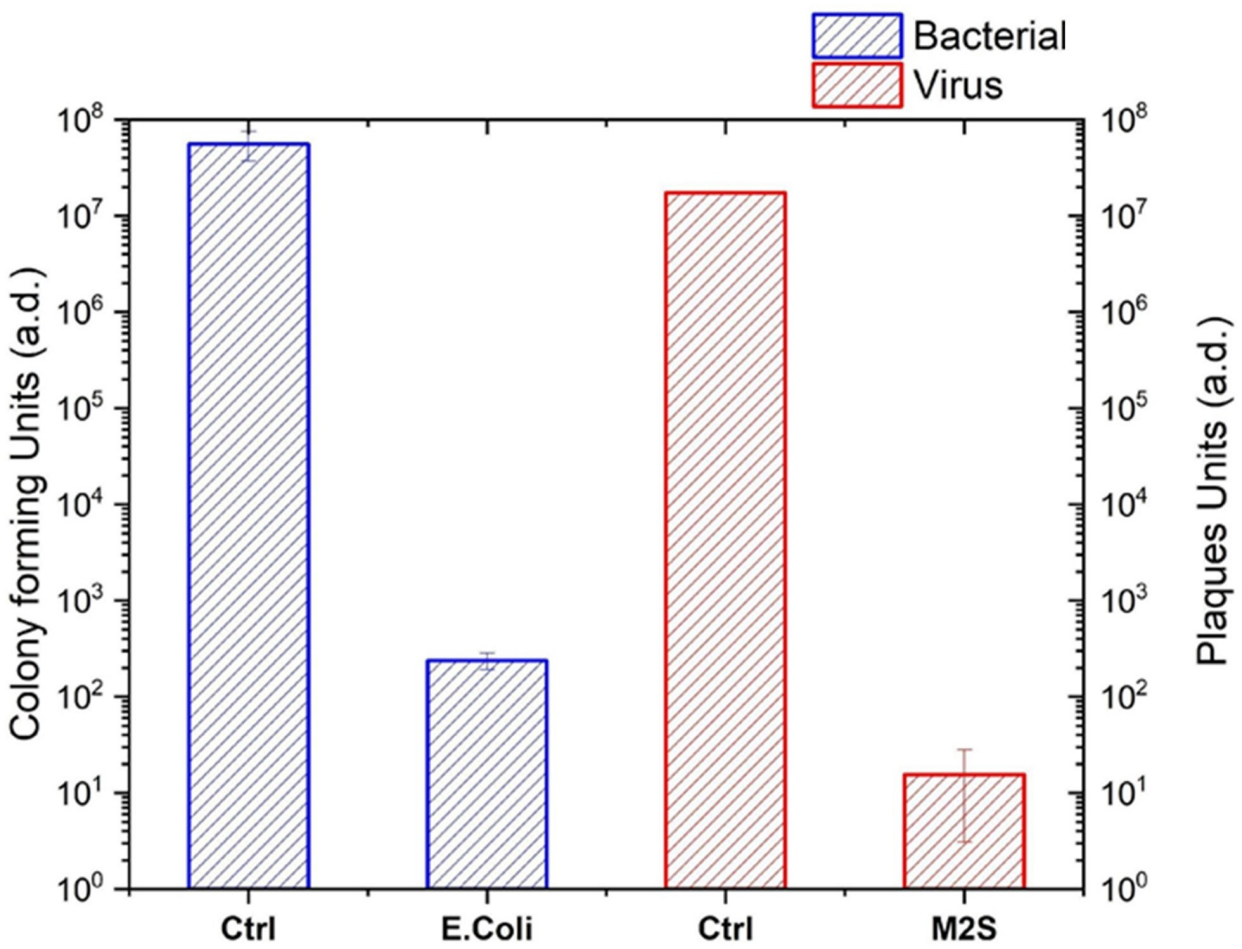
End-point treatment decontamination efficiencies for bacterial (*E*.*Coli*) and virus (MS2 bacteriophages) in terms of colony forming or plaques units after 15 minutes of masks exposures to the plasma treatment.

Untreated control masks showed an average recovery of *E*.*coli* about 5x10^7^ bacteria /mL. After 15 minutes of plasma treatment the average number of bacteria recovered from the mask was 3.2x10^2^ bacteria/mL which results in a Log-reduction value > 5 in the bacteria number (Fig 4). For bacterial models, this is in line with killing efficacy reported in literature for atmospheric pressure plasma systems over flat, non micro-porous substrates [50, 51]. This result implies a good diffusivity of the plasma sterilising agents within the mask microporosity, in our experimental conditions. Since inactivated spores are also recovered from the deepest mask layers it can also be assumed, via a line-of-sight argument, that the role of UV photons in decontamination and inactivation might be negligible. Considering the modest increase in substrate temperature it is possible to suggest that the plasma–biomaterials interaction driving the decontamination process is carried out mainly by long lived chemical species able to diffuse along the carrier gas stream and diffuse through the substrate micro-porosity. The same experiment performed using a viral model (bacteriophages MS2) showed even more encouraging results. In this case, the average recovery from the control gave a 2x10^7^ PFU/mL while after plasma treatment (applying the same conditions) the average recovered PFU/mL were less than 2x10^1^ units. The average Log-reduction of plaques units achieved in this experiment was Log > 6. For comparison with real applications requirements, efficacy data recommendations from EPA for decontamination on hard (non-microporous) surfaces require for a given sterilising agent to be able to reach a Log-6 bioburden reduction for a process lasting for maximum 10 minutes [52].

**Fig 5:**
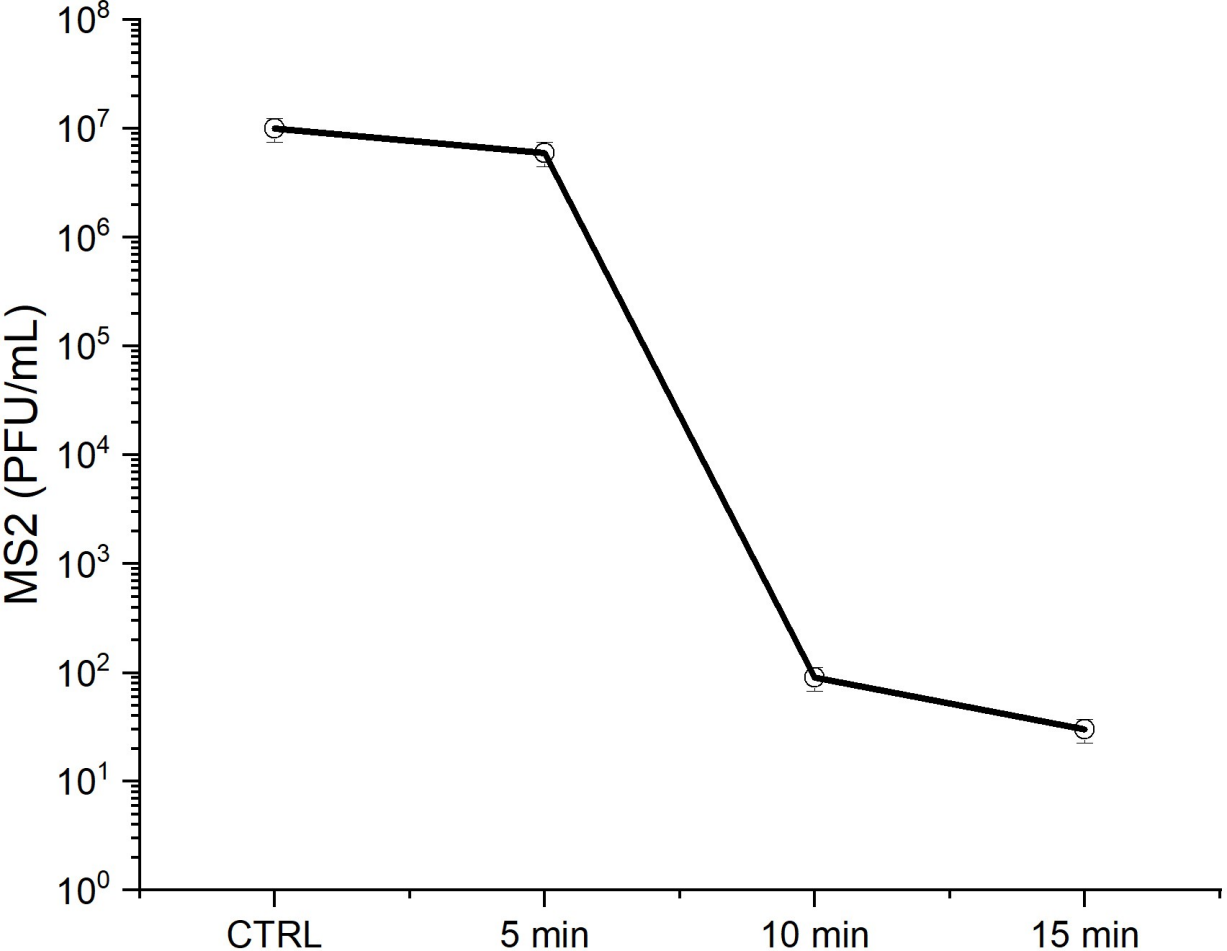
MS2 bacteriophages plaques forming units/mL count after plasma treatment (different time points).

To evaluate the kinetic of viral models inactivation on respiratory masks, a suspension of MS2 was spotted on the mask and then treated at three different time points (5 min, 10 min and 15 min). The infectivity of treated bacteriophages was assessed by a determination of PFU count. The plasma treatment at the endpoint reduced the PFU/mL from 10x10^7^ to 10x10^1^ PFU/ml (Fig 5). As expected PFU/mL decreased with increasing plasma exposure time but, notably, the rate at which viruses are eliminated from the mask fibres changes with time. The three slopes curve (in a log-linear plot) indicates the presence of different time constants, or equivalently different interaction mechanisms, governing the decontamination kinetics with the plasm-virus-masks system. From these data alone, a direct interpretation of the interaction mechanisms is complicated by the overlap of different channels involving the plasma-generated sterilising species diffusing with the mask tissue microporosity and the interaction between plasma species and the biomolecular structure of the virus [53]. From the data in Fig 5 we can observe an initial slow phase lasting till about 5 min, followed by a faster decay lasting till about 10 min from the start of treatment and finally another slow phase (although with a different slope with respect to the initial one). A bacteriophage population reduction greater than 6 orders of magnitude is achieved within 10 and 15 minutes of plasma treatment.

**Fig 6:**
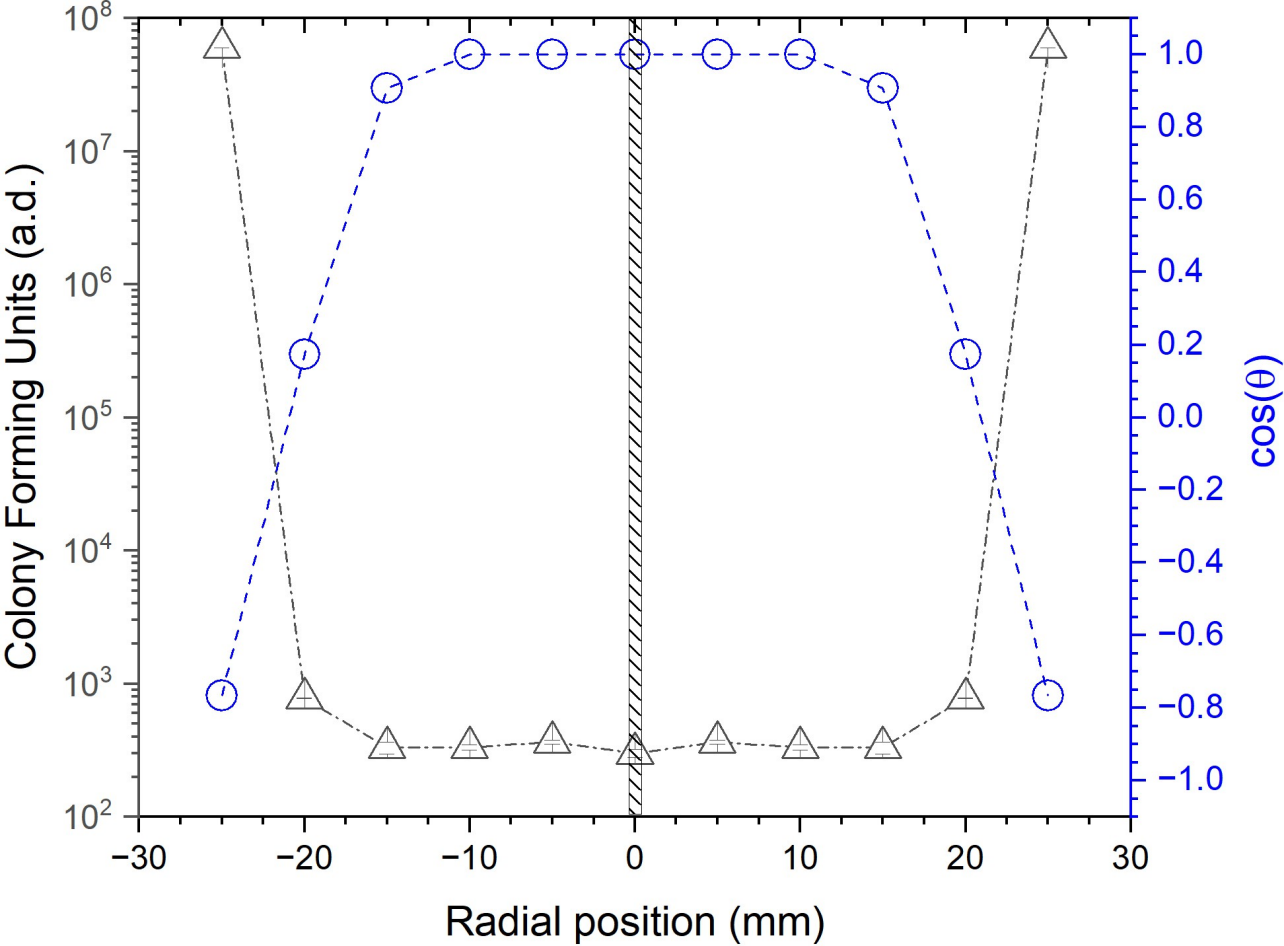
Left axis (black), colony forming units recovered from respiratory face mask filter material after 15 mins exposure to the plasma treatment, as a function of the radial position from the plasma jet vertical. Right axis, in bleu, water contact angle expressed as cos(θ) according to the Young formula after 15 mins exposure to the plasma treatment as a function of the radial position from the plasma jet vertical. For comparison the physical extension of the plasma jet aperture is indicated with the black striped area around radial position zero.

Our plasma system prototype comprises a single APPJ element but in principle this kind of system is easily scalable for processing large 2D surfaces by arranging several individual plasma elements in arrays or batteries [54]. For a given treatment an important parameter for the engineering of such arrays is the area over which a single plasma jet can impart the intended surface modifications, in our case decontamination of viral/bacterial load on respiratory masks substrates. This parameter defines the spacing between individual plasma sources in a battery and the total number of sources (and power, gas etc.) needed to process a given item’s surface area. The radial profiles of induced plasma modifications, or equivalently the area of effectiveness of an individual plasma jet source, were investigated by monitoring the biocidal effects of pathogens deposited on the treated substrate. The experiment was conducted comparing flat, non porous (agar gel) and porous (mask materials) materials, then measuring the presence of pathogens after the treatment at an increasingly larger distance from the vertical of the plasma nozzle. For all experiments a constant nozzle-surface distance of 2 mm was maintained. The control experiment using flat surfaces was performed by *E*. *Coli* colony counts on an agar gel plate after exposure to He/O_2_ plasma for different times and measuring the corresponding inhibition zone (see S5 Fig in S1 File). The test on porous mask materials was performed by recovering bacteria from the contaminated mask layers (exposed for a fixed treatment time) at positions located at increasing radial distances from the plasma nozzle vertical, according to the protocol described in the materials and methods section. In the first control experiment, *E*.*coli* was plated on LB agar plates and exposed to the plasma jet stream, the results indicate a significant reduction of bacteria growth around the zone of exposition (S6a, S6b Fig in S1 File). The inhibition area was calculated from photographic images and it was found that 300 seconds of plasma treatment were sufficient to significantly reduce the bacteria growth (p<0.0001) at the plate center. Additionally, exposure times longer than 300 s further increase the inhibition zone in a time dependent manner (S6b-S6d Fig in S1 File). After 15 min of plasma exposure the inhibition zone on flat surfaces reached a circle of 4 cm radius. The same experiment was then repeated on a large section of respiratory masks contaminated with bacteria. The whole mask was contaminated and then, after treatment, the surviving bacteria were recovered by smaller square sections (25 mm^2^) located at 5 mm distance (centre-to-centre) from each other. Several specimens collected at increasing distance from the plasma jet vertical axis (position zero) were evaluated. On the same small specimens the H_2_O-CA immediately after plasma treatment was measured in parallel with the bacterial count. Results of these combined experiments are summarised in Fig 6. Data shows that after 15 minutes of plasma exposure respiratory mask materials decontamination factor of, at least, Log5 is achieved against bacterial spores over a circular area covering radial locations distant up to 22.5 mm from the point of application (ca. 15.9 cm^2^). Outside this circular region, the bacterial viability sharply increases back to values comparable with the control. Simultaneously, the wetting character of the surface changes from hydrophobic (water contact angle, cos(θ_H2O_) = - 0.8, according to Young Formula) to hydrophilic (cos(θ_H2O_) = 1.0) over a circular area quite precisely overlapping with to the decontaminated region as verified by the CFU counts experiments. The grafting of plasma generated ROS to the polypropylene mask surface and the subsequent surface peroxides formation is supported by ATR-FTIR measurements shown in Fig 2 and can be thought as the likely mechanisms driving the wettability character modification. The same ROS, reaching biomolecules and microorganisms located in the porous mask materials can therefore also induce enough oxidative stress to cause their inactivation. These results confirm the strong link between the ROS species in the plasma stream, their gas-phase diffusion in the respiratory mask filter materials and the efficacy of the plasma treatment for bacterial and viruses decontamination. In Fig 6, for comparison the physical width of the plasma jet aperture (1 mm wide) is indicated with the black striped area around radial position zero, thus obtaining for our laboratory prototype a ca.1600:1 ratio between the size of the plasma jet and the extension of the effectively treated area.

### Environmental impact considerations for potential applicability

This study focuses on exploring the possibility of safe re-use of respiratory masks (type FPP2 and FPP3, EU standards) protective equipment for airborne pathogens transmission by applying a decontamination treatment based on atmospheric pressure plasma processing. In the previous paragraph, data about decontamination efficacy and mask material modifications were presented. During a public health crisis like the COVID-19 crisis, decontamination and re-use of medical PPE could represent both a short- and a long-term emergency response. Short-term, a technological solution allowing hospitals and health-care centres to manage in-house, safe re-use of PPE would greatly increase their resilience and preparedness during peak supply crisis without relying on expensive preventive storage solutions. A mature PPE re-use technology will allow health organisations to maintain their economical favourable just-in-time supply chain structure while at the same time guaranteeing the capability to adequately protect their most critical asset, the health operators, and thus the organisation capacity of operating at full or near-full capacity under stress scenarios.

Long-term, considering PPE alone the enormous investment of resources used to contrast COVID19 pandemic poses a question of sustainability and environmental impact, since during the pandemic billions of PPE units per year were incinerated as medical waste. A re-use approach must be designed considering the whole item life cycle, balancing the decreased impacts in terms of manufacturing, packaging, transport and end-of-life with the additional resources utilised to carry out the regeneration process itself.

A thorough analysis of plasma-decontaminated masks life-cycle based on the data produced in this study has been carried out in parallel with and based on the experimental data obtained in this study [40]. In particular, the environmental impacts of the single-use and plasma-decontaminated masks scenarios were calculated. The simulations assess that the re-use and decontamination scenarios results in an overall higher environmental impact than the single use scenario. Analysing the contributions of materials and consumables on the single impact categories it results that the decontamination process exhibits the highest impacts due to the carrier helium gas consumptions as process gas and electrode coolant. He utilisation accounts for 95% of the impact in most of the impact categories (i.e. climate change, ozone depletion, particulate matter, photochemical ozone formation, acidification, water use, land use, fossil and mineral resource use, human- and eco-toxicity). Helium is a widespread choice as carrier gas in APPJ processing of thermos-labile materials because helium plasma tends to show more diffusivity compared with the argon plasma, moreover it shows lower electron density and gas temperature but larger volume, in contrast Argon gas is more economical. Therefore, for many thermally sensitive plasma applications, especially applications in biomedicine, a diffusive and homogeneous He discharge is preferred since it provides an uniform treatment and is capable of avoiding damage to sensitive tissues and materials [26, 55]. Additional scenarios simulating reduced He consumptions for our decontamination experiments were explored to assess possible impact mitigation routes, in particular: i) He replacement with Ar or N_2_, ii) reduced He flow (alternative cooling strategy) and iii) He recirculation and re-use. These conclusions highlight the importance of keeping a broad eyesight and including also environmental impact considerations during the development and optimization of novel technological solutions.

Recently other similar studies appeared in literature, analysing other mask decontamination technologies in view of their environmental performance, namely autoclave based steam sterilization [56] and hydrogen-peroxide based sterilization [57].

## Conclusions

This study assessed the feasibility of an atmospheric pressure plasma approach for the decontamination of disposable filtering face piece respirators (FFR) or respiratory masks commonly used in hospital settings. A lab-scale demonstrative prototype was realised and tested directly over mask materials contaminated with different types of bioburden, including viruses (MS2 bacteriophages viral models). Plasma treatment parameters were chosen in order to minimise modifications of the physical-chemical properties of mask filtering materials induced by substrate heating and chemically reactive species flow. Morphology, structure and surface chemistry of the masks PP fibres show only minor modifications upon cyclic treatment and surface oxidation due to plasma exposure resulted to be partially transitory. The efficacy of plasma-based face masks decontamination was demonstrated for both viruses and bacteria, achieving a Log6 reduction of the viral load initially present within the mask structure after 15 minutes of plasma exposure, while similar test on bacterial contamination yielded a Log5 reduction within the same time frame. The effluent of the plasma jet proved to be an efficient way of transferring the sterilising chemistry well within the deep layers of the respiratory filters and achieve high levels of decontamination. The observations carried out in this research point to long-lived reactive species as the most likely channel dominating the plasma-biomolecules interaction, either via oxidative damage and/or biomaterials etching, and thus driving the decontamination process. In conclusion, a few considerations about the plasma decontamination process environmental impact (as compared to respiratory masks single use) pointed out which critical process parameters (i.e. He consumption) must be optimised in order to minimise resources use and emissions.

## Supporting information

S1 File Supporting information file. Supporting data S1-S6 Figs are available this file.
(DOCX)
