# Supplementary material for: Atmospheric pressure plasma jet for respiratory face masks decontamination and re-use: Considerations on microbiological efficacy, material impact and product lifecycle
Source: PLoS One. 2025 Jan 23;20(1):e0313041. doi: 10.1371/journal.pone.0313041 (PMC11756767; doi:10.1371/journal.pone.0313041)
Supplement: S1 File — Supporting data S1-S6 Figs are available this file. (DOCX) [file pone.0313041.s001.docx]

**Atmospheric pressure plasma jet for respiratory face-masks decontamination and re-use. Considerations on microbiological efficacy, material impact and product lifecycle.**

Diletta Scaccabarozzi^§^, Jessica Ponti^§^, Sabrina Gioria^§^, Dora Mehn^§^, Taija Sinkko^§^, Fulvio Ardente^§^, Francesco Fumagalli^§,*^.

^§^ European Commission, Joint Research Centre (JRC), Ispra, Italy;

* Corresponding author: [francesco-sirio.fumagalli@ec.europa.eu](mailto:francesco-sirio.fumagalli@ec.europa.eu)

**Supporting Info**


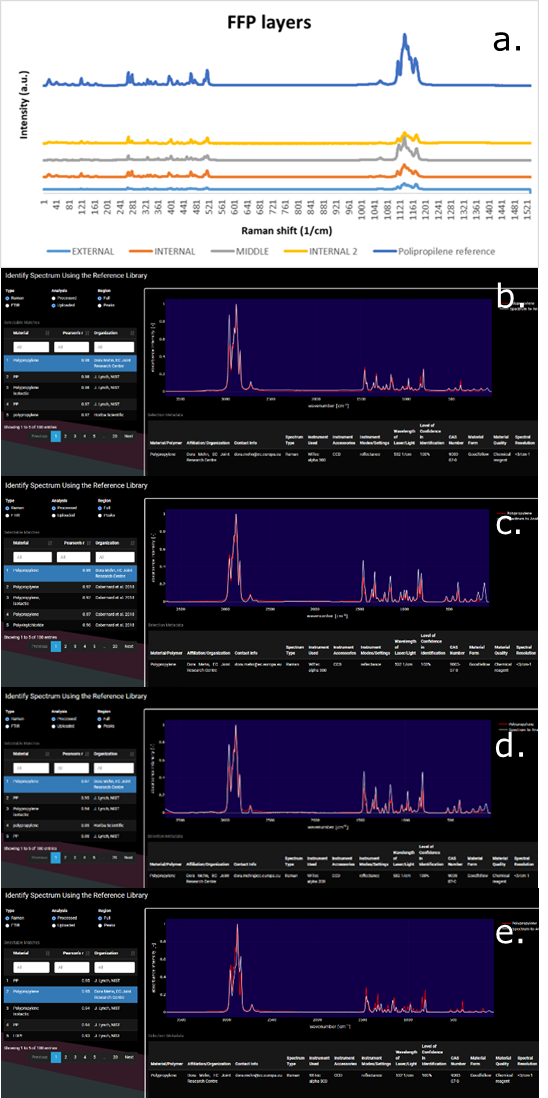


*Figure SI1*. a) micro-Raman analysis of four different non-vowen layers comprising mask ARIA. Spectral assignments were verified using our internal reference library for polymers spectra and using the online tool: [www.openanalysis.org/openspecy](http://www.openanalysis.org/openspecy) (b-e, for each layer starting from the outer one)


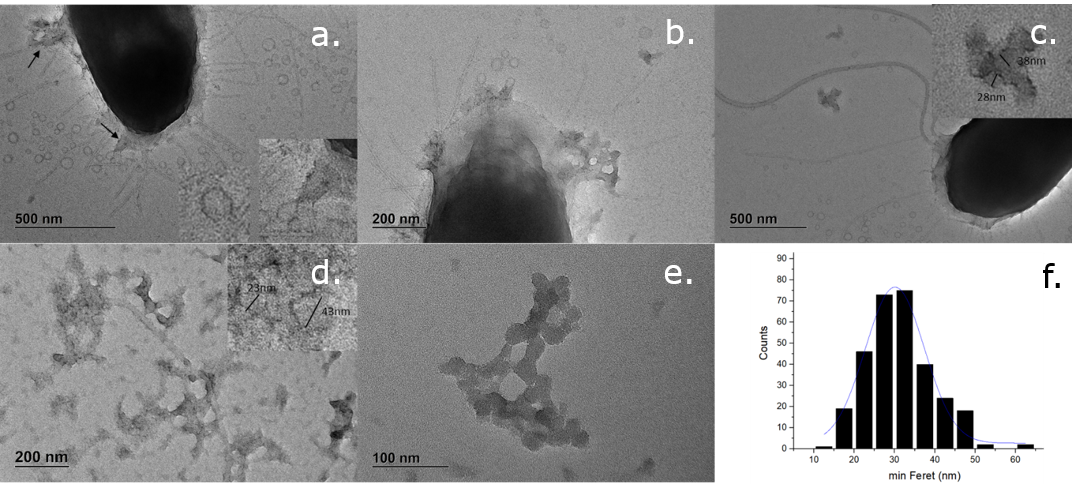


*Figure SI2*. Bacteriophages appear agglomerated during their release from bacterial wall, as a sort of gemmation, individually distributed around bacteria and also attached to pili as a defined growth process. (A-C) Once free in culture medium, they tend to agglomerate-aggregate also forming bigger particles of around 60nm where it is possible to distinguish each single phage. (D-E) This sticky behaviour is also observed once the ‘purified’ MS2 suspension is observed. MS2 bacteriophages show spherical shape with mean of min Feret size 30,1±7,3nm. (F)


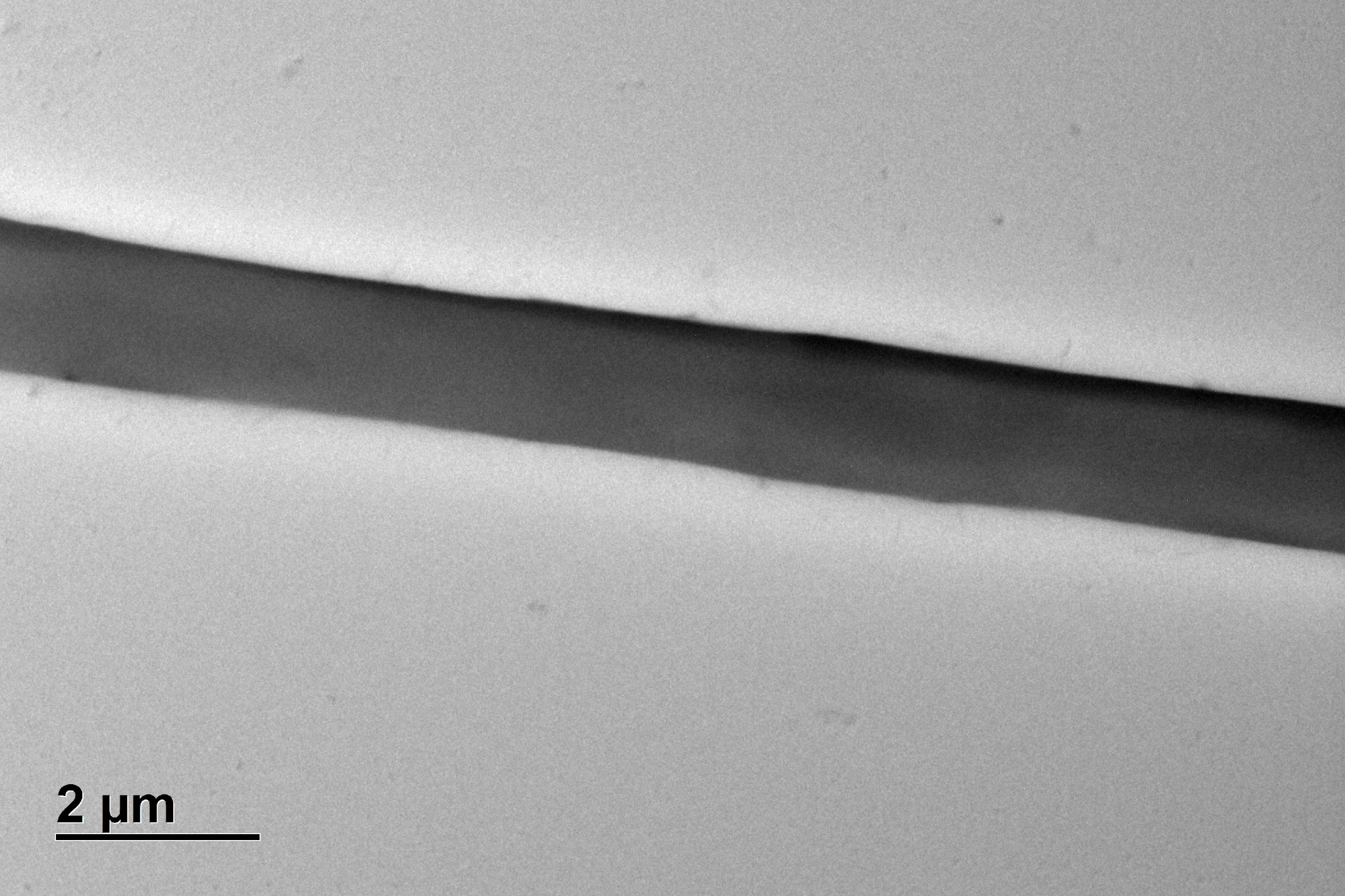

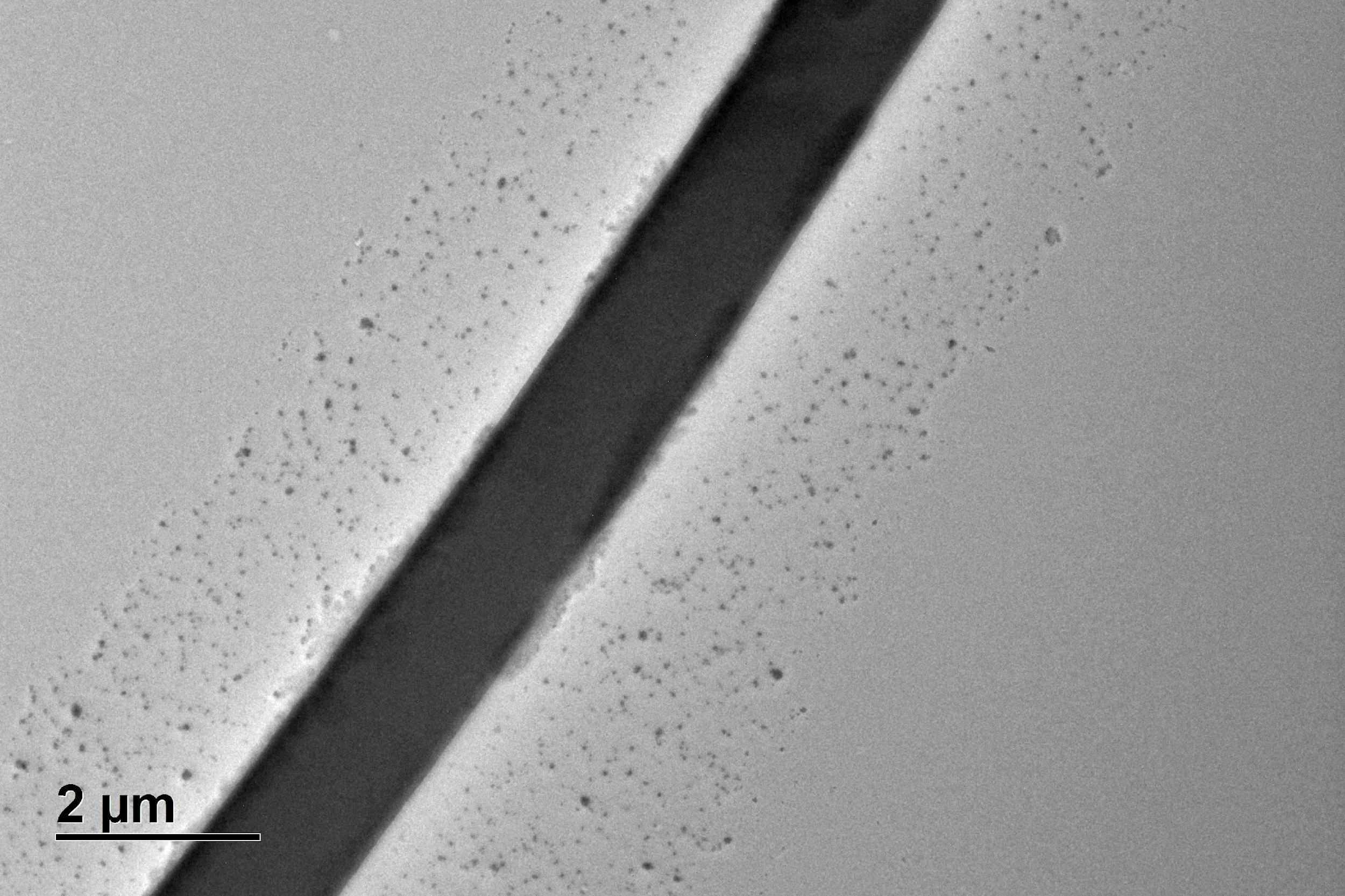

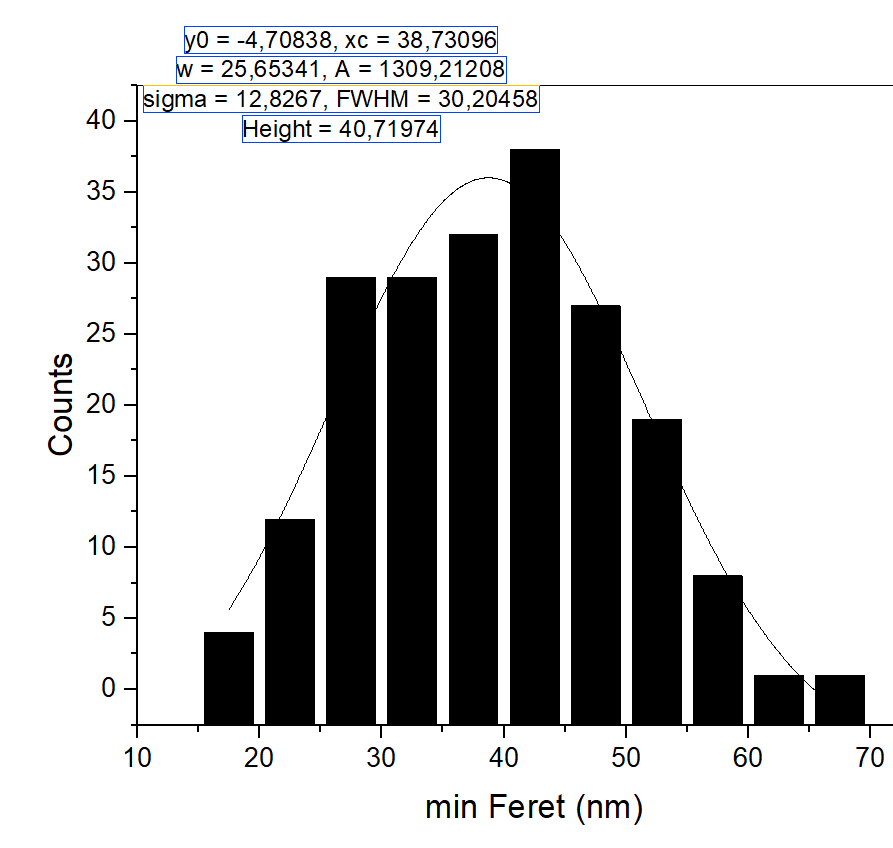


*Figure SI3* Representative image of a Control (unexposed) mask fiber and mask fiber incubated to MS2 and corresponding size distribution of MS2 directly on mask.


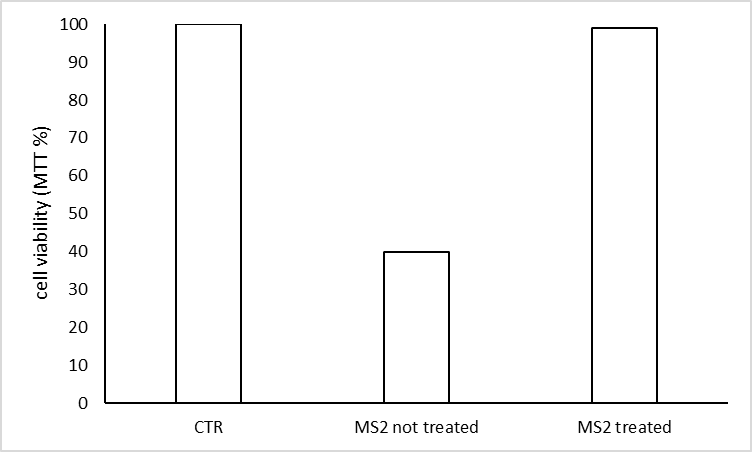


*Figure SI4*. Cell viability data for cultures exposed to MS2 bacteriophages exposed or not to the plasma discharge.


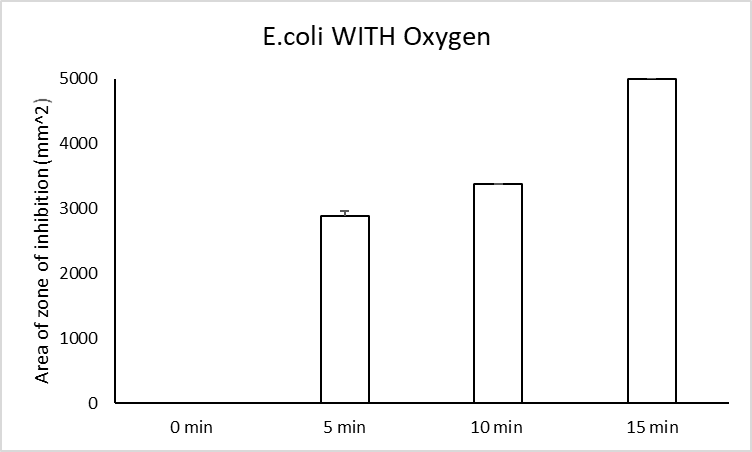


*Figure SI5*. Calculated bacterial growth inibithion zones as a function of plasma treatment time.


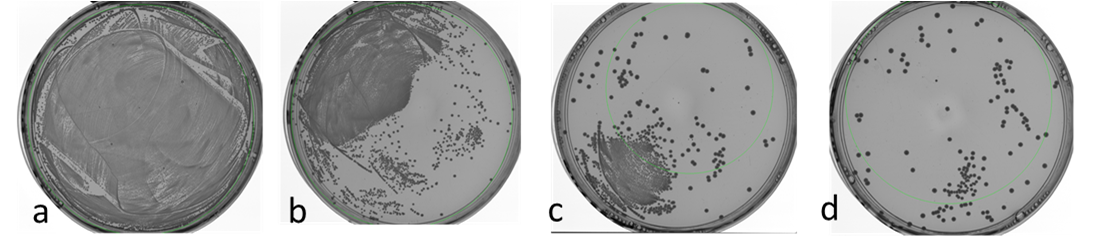


*Figure* SI6. Bacterial growth inhibition zones on Agar-coated plates after plasma treatment. a) control, 0 min. b) 5 min. c) 10min and d) 15 min
